# Supplementary material for: MICU1 drives glycolysis and chemoresistance in ovarian cancer
Source: Nat Commun. 2017 May 22;8:14634. doi: 10.1038/ncomms14634 (PMC5477507; doi:10.1038/ncomms14634)
Supplement: Supplementary Information — Supplementary Figures and Supplementary Tables [file ncomms14634-s1.pdf]

(A) (B)

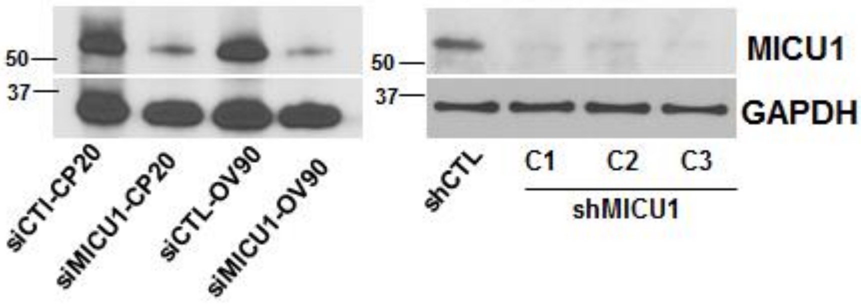

Supplementary Figure 1. Efficient silencing of MICU1. (A) 5X10^5 CP20 or OV90 cells were transiently transfected with MICU1 siRNA or control siRNA using HiPerfect transfection reagent and post 48h the extent of MICU1 silencing was determined at the protein level by immunoblotting for MICU1. (B) Stably transfected OV90 cells with lentiviral shRNA for MICU1 (subjected to puromycin selection) were tested for knockdown efficiency in three different clones (C1, C2, C3) selected by immunoblotting for MICU1. GAPDH was used as a loading control.

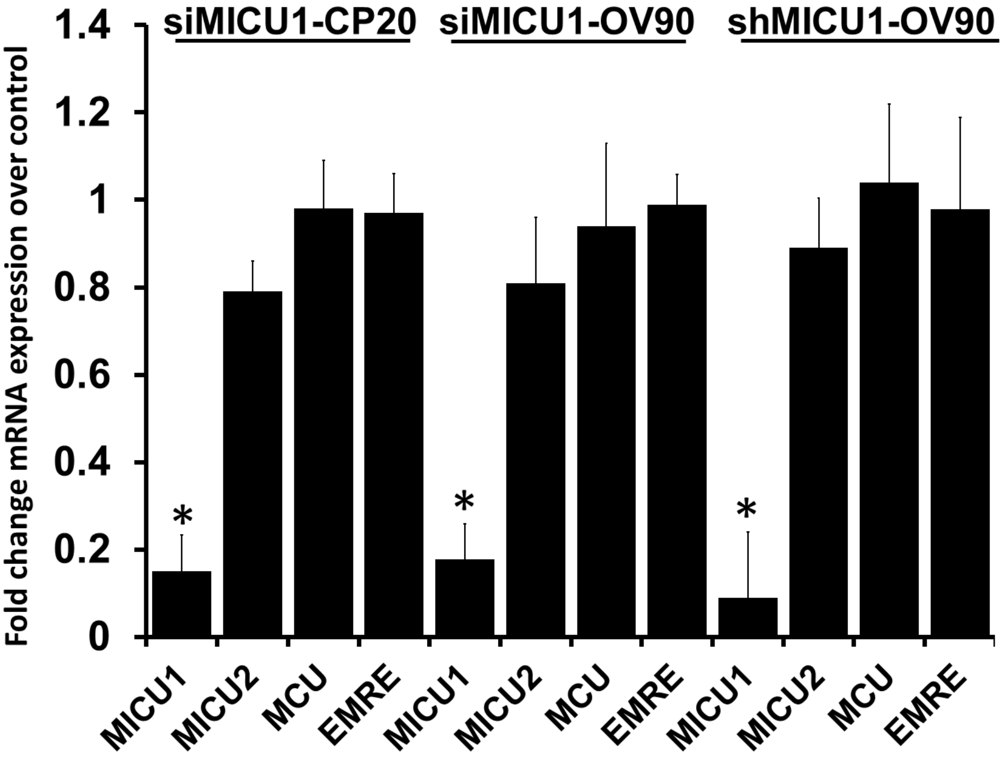

Supplementary Figure 2. Silencing of MICU1 has minimal effect on the expression of other MCU components. Relative mRNA expression of genes involved in the MCU component (MICUI, MICU2, MCU, EMRE) was determined in CP20 and OV90 cells. MICU1 gene was silenced using siRNA and shRNA approach as described in supplementary figure 1 and cells were harvested after 48 hours post siRNA transfection incase of transient silencing of MICU1. Data is presented as fold change over siCTL or shCTL where applicable. Gene expression was normalized against 36β4. All the experiments were repeated independently at least 3 times and in triplicate. Data are expressed as mean ± standard deviation (SD). *, $P<0.05$ considered statistically significant when compared to the respective controls, using two-sided Student's t-test with unequal variances.

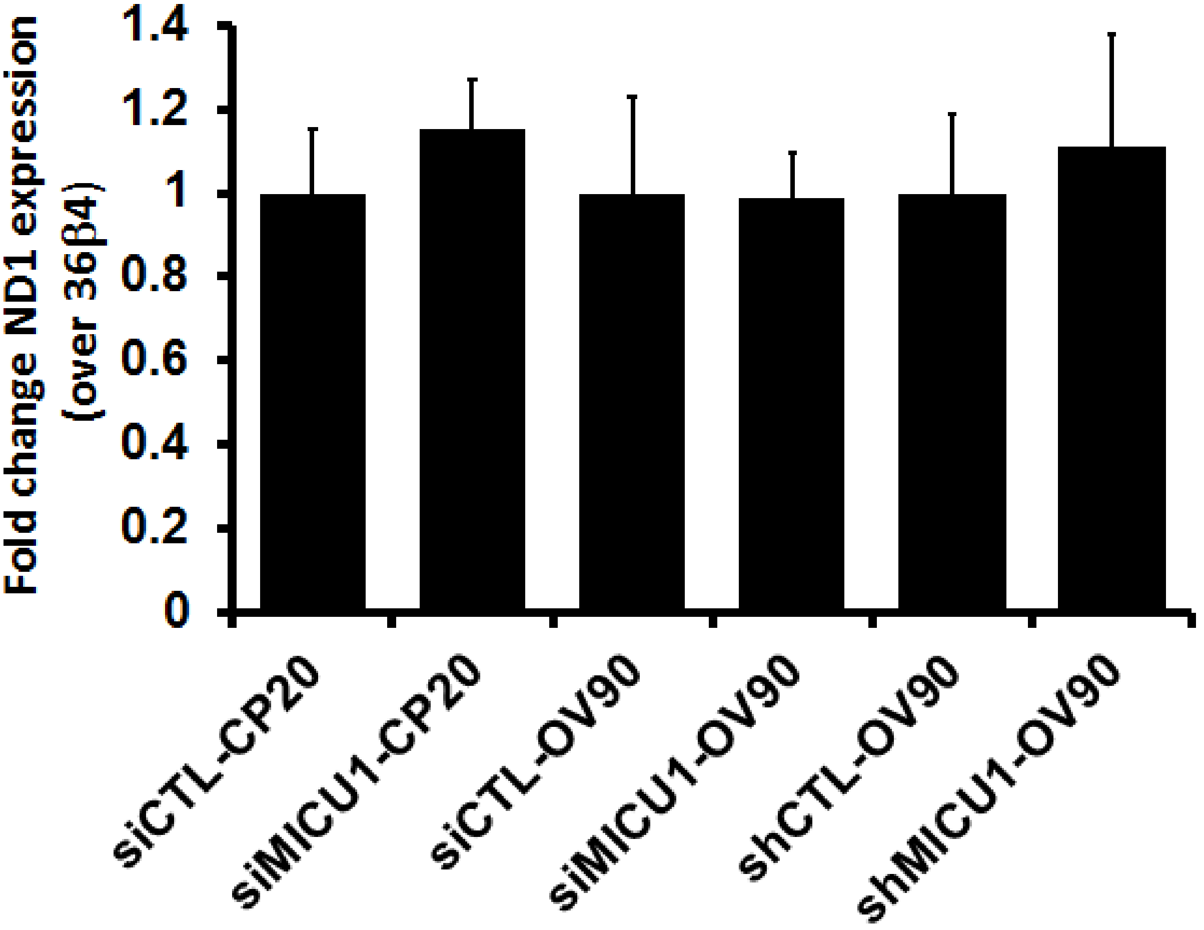

Supplementary Figure 3. Mitochondrial copy number remains unchanged upon MICU1 silencing. Total genomic DNA was isolated with the QIAamp kit (Qiagen). Mitochondrial copy number was evaluated based on the ratio of the nuclear DNA to mitochondrial DNA Briefly, abundance of nuclear encoded 36B4 was compared to mitochondrial encoded ND1 (Complex I). Gene expression was determined by quantitative PCR as described previously. All the experiments were repeated independently at least 3 times and in triplicate. Data are expressed as mean ± standard deviation (SD). Data is not significant when compared to the respective controls, using two-sided Student's t-test with unequal variance.

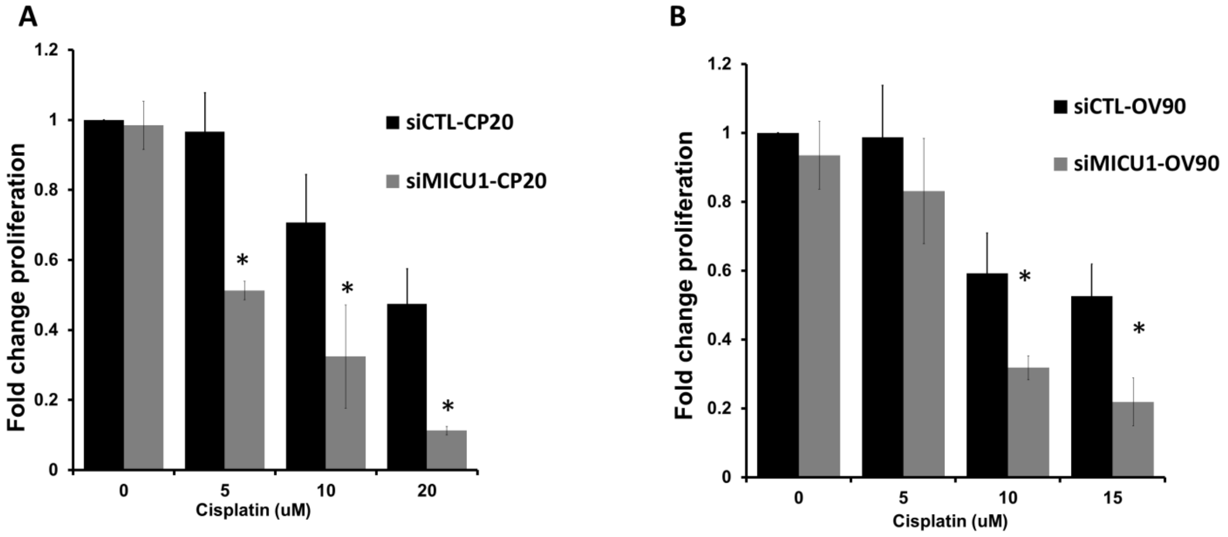

Supplementary Figure 4. MICU1 silencing sensitizes CP20 and OV90 cells to cisplatin. BrDU incorporation during DNA synthesis in replicating cells was assayed to determine cellular proliferation. To assess the proliferation, experiments were performed in cells transfected with negative siRNA (siCTL) or MICU1 siRNA (siMICU1) in CP20/OV90 cells for 48h and then equal number of $3X10^3$ cells were re-plated in 96-well plates. Cells were treated with cisplatin at the indicated concentrations for 24h. Cell proliferation was evaluated in the control (MICU1 expressing) or MICU1 silenced cells and data is represented as mean $\pm$ SD with values of vehicle treated siCTL transfected cells set to 1.. Comparisons between two groups treated with same dose of cisplatin were evaluated. *, $P<0.05$ was statistically significant when compared to respective control. Two-sided Student's t test with unequal variance was used for statistical analysis. All the experiments were repeated independently at least 3 times and in triplicate.

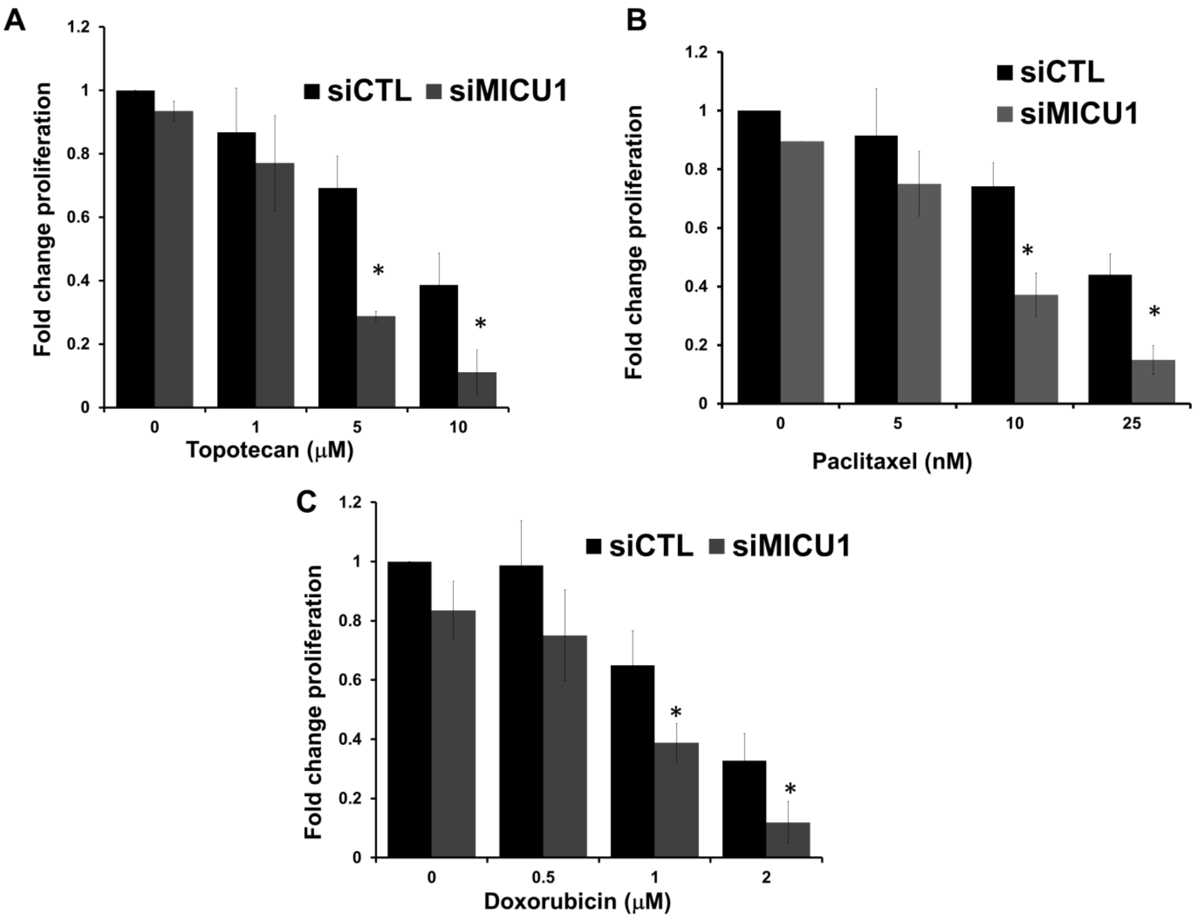

Supplementary Figure 5. MICU1 silencing sensitizes CP20 cells to different chemotherapeutic drugs. CP20 cells were transfected with siCTL or siMICU1 for 48h and equal number of $3X10^3$ cells were re-plated in 96-well plates. Cells were treated with Topotecan, Paclitaxel and Doxorubicin at the indicated concentrations and cell proliferation was determined using CyQUANT® NF Cell Proliferation Assay after 48h by measuring the fluorescence (excitation ~485 nm and emission ~530 nm). Data is represented as mean $\pm$ SD with values of vehicle treated siCTL transfected cells set to 1. Comparisons between two groups treated with same dose of the drug were evaluated. *, $P<0.05$ was statistically significant when compared to respective control. Two-sided Student's t test with unequal variances was used for statistical analysis. All the experiments were repeated independently at least 3 times and in triplicate.

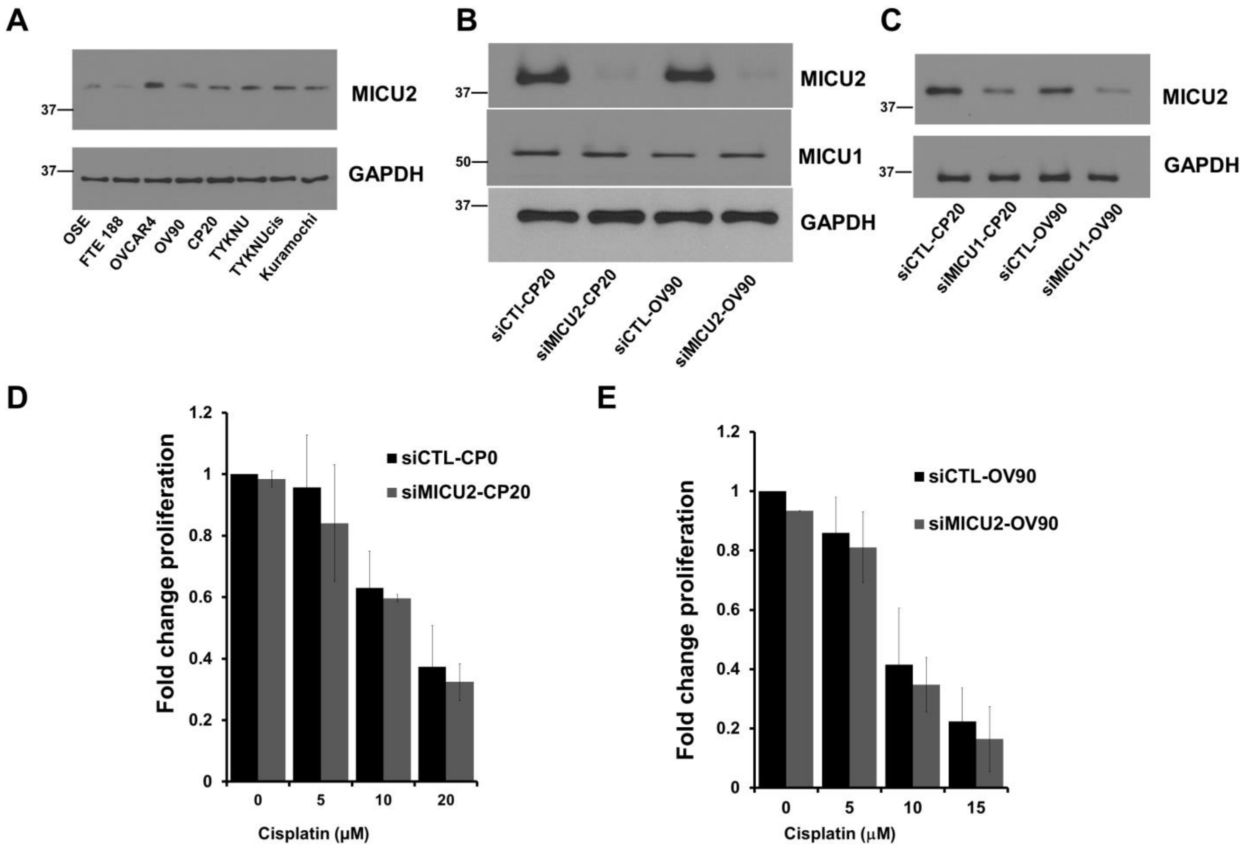

Supplementary Figure 6. MICU2 does not contribute to chemosensitization. (A) Protein Expression of MICU2 in a panel of ovarian cancer cell lines was determined by immunobloting using MICU2 antibody (Abcam #ab101465) and GAPDH was used as a loading control. (B) Protein expression of MICU2 and MICU1 was determined by immunoblotting in CP20 and OV90 cells either transfected with scrambled siRNA (siCTL) or MICU2 siRNA, GAPDH is used as a loading control. (C) Protein expression of MICU2 in MICU1 silenced cells was determined by immunobloting, where GAPDH was used to indicate loading control. (D) CP20 cells or (E) OV90 cells were transfected with siCTL or siMICU2 for 48h and cells were re-plated in 96-well plates. Cells were treated with increasing concentrations of cisplatin and after 48 h cell proliferation was determined using CyQUANT® NF Cell Proliferation Assay by measuring the fluorescence (excitation ~485 nm and emission ~530 nm). Data is presented as fold change with scrambled siRNA transfected -untreated cells considered as 1. Comparisons was done between siCTL and siMICU2 transfected cells receiving same dose of cisplatin treatment. Data are expressed as mean ± SD. Data is not significant when compared to the respective controls, using two-sided Student's t-test with unequal variance.

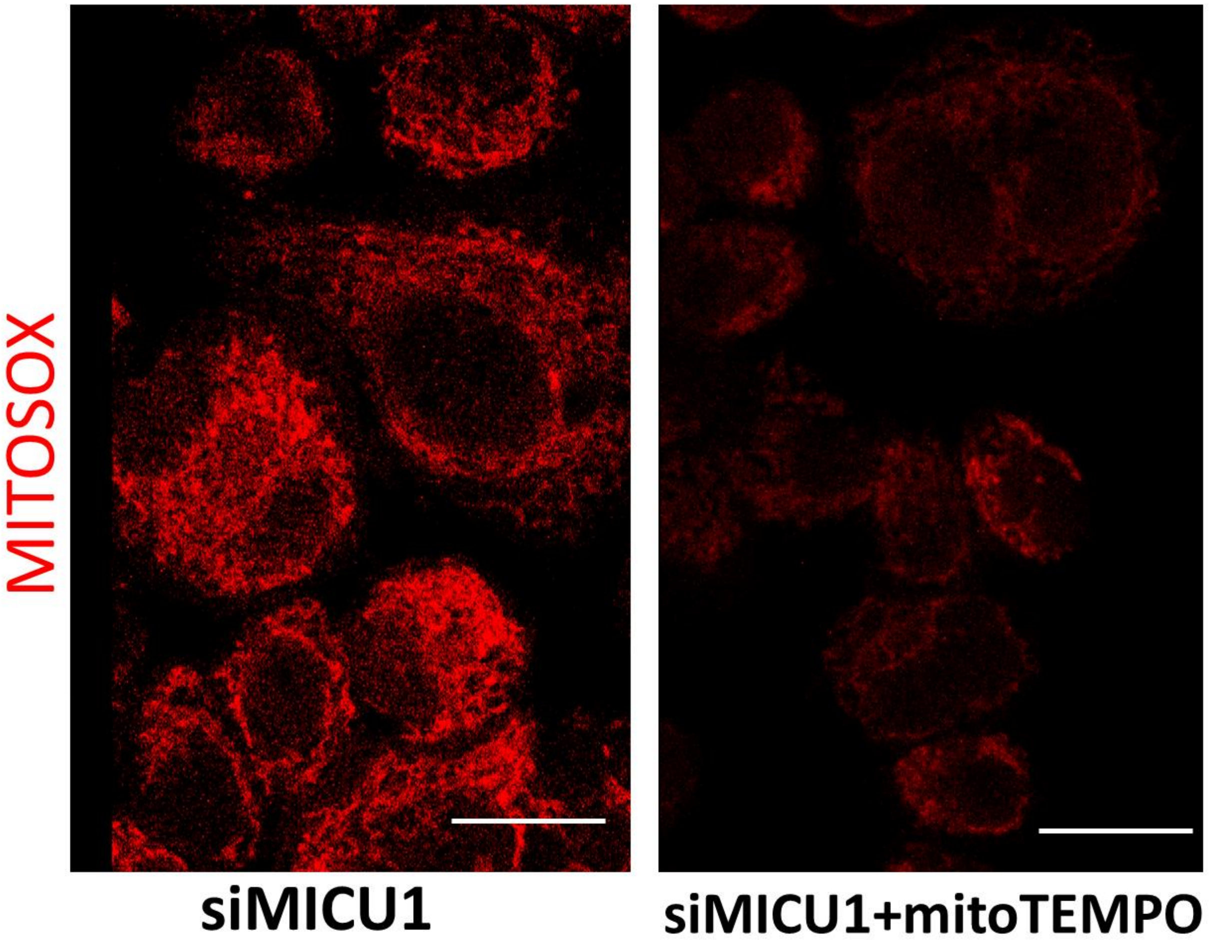

Supplementary Figure 7. Lowering of mROS by mitoTEMPO. 2X10^4 CP20 cells were grown on coverslips and transfected with siRNA against MICU1 for 48 h. Transfected cells were further stained with 1 μM MitoSOX (red) dye for 20min with or without preincubation with mitoTEMPO (10μM). Live cell imaging was performed with Zeiss Axio-Observer Z1 (Göttingen, Germany) using 510nm excitation and 580nm emission wavelengths. White bar indicates scale bar = 10μm.

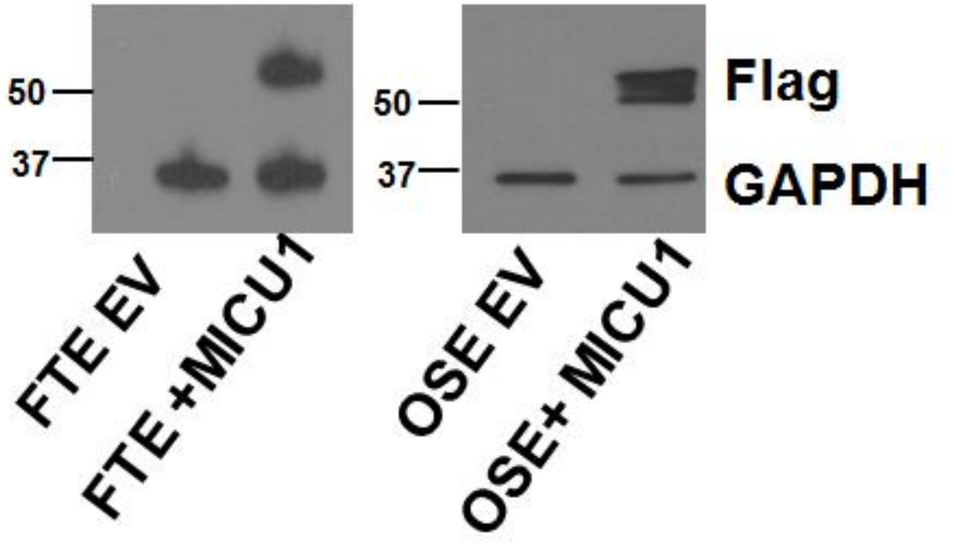

Supplementary Figure 8. Ectopic expression of MICU1 in FTE188 and OSE cells. Flag tagged MICU1 (1µg) was transfected in 10^6 cells of Fallopia Tube Epithelial, FTE 188 and Ovarian surface epithelial, OSE cells using Lipofectamine 3000 transfection reagent as per manufacturer's protocol. 24h post transfection, protein expression of exogenous MICU1 was determined by immunoblotting with Flag antibody. GAPDH was used as loading control.

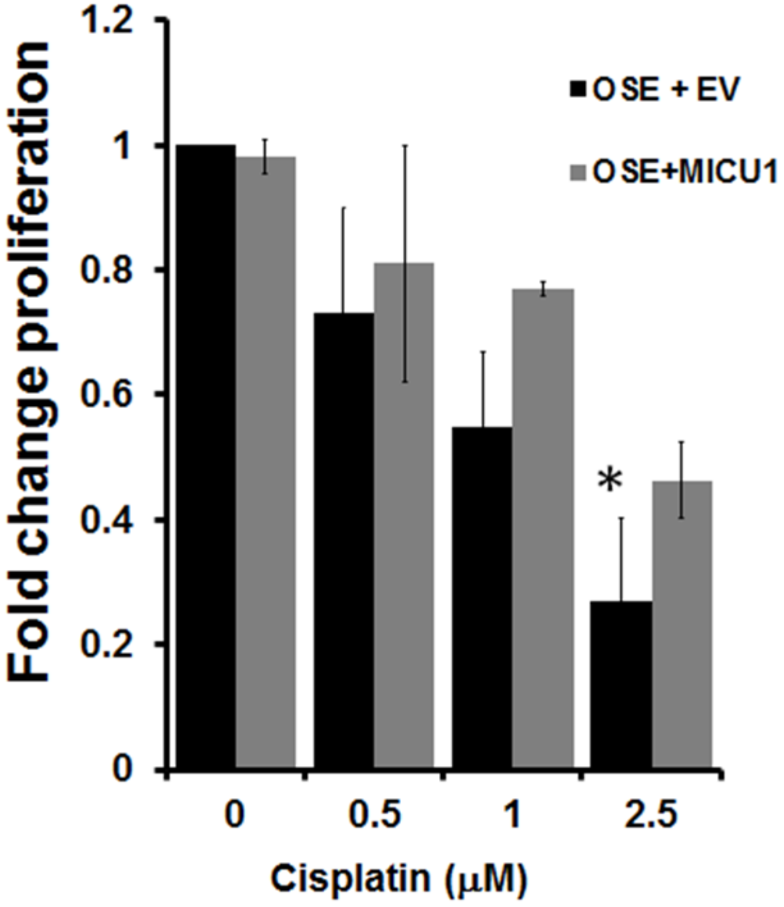

Supplementary Figure 9. Ectopic expression of MICU1 in OSE cells. Flag tagged MICU1 (1μg) or empty vector (1μg) were transfected in 10^6 cells of Ovarian surface epithelial, OSE cells using Lipofectamine3000 transfection reagent as per manufacturer's protocol. 24 h post transfection, cells were re-plated in 96-well plates. Cells were treated with increasing concentrations of cisplatin and after 48 h cell proliferation was determined using CyQUANT® NF Cell Proliferation Assay by measuring the fluorescence (excitation ~485 nm and emission detection ~530 nm). Data are expressed as mean ± SD. Statistical significance was set at $P < 0.05$, and indicated by *. For comparisons among more than two groups, ANOVA was performed with Bonferroni's correction.

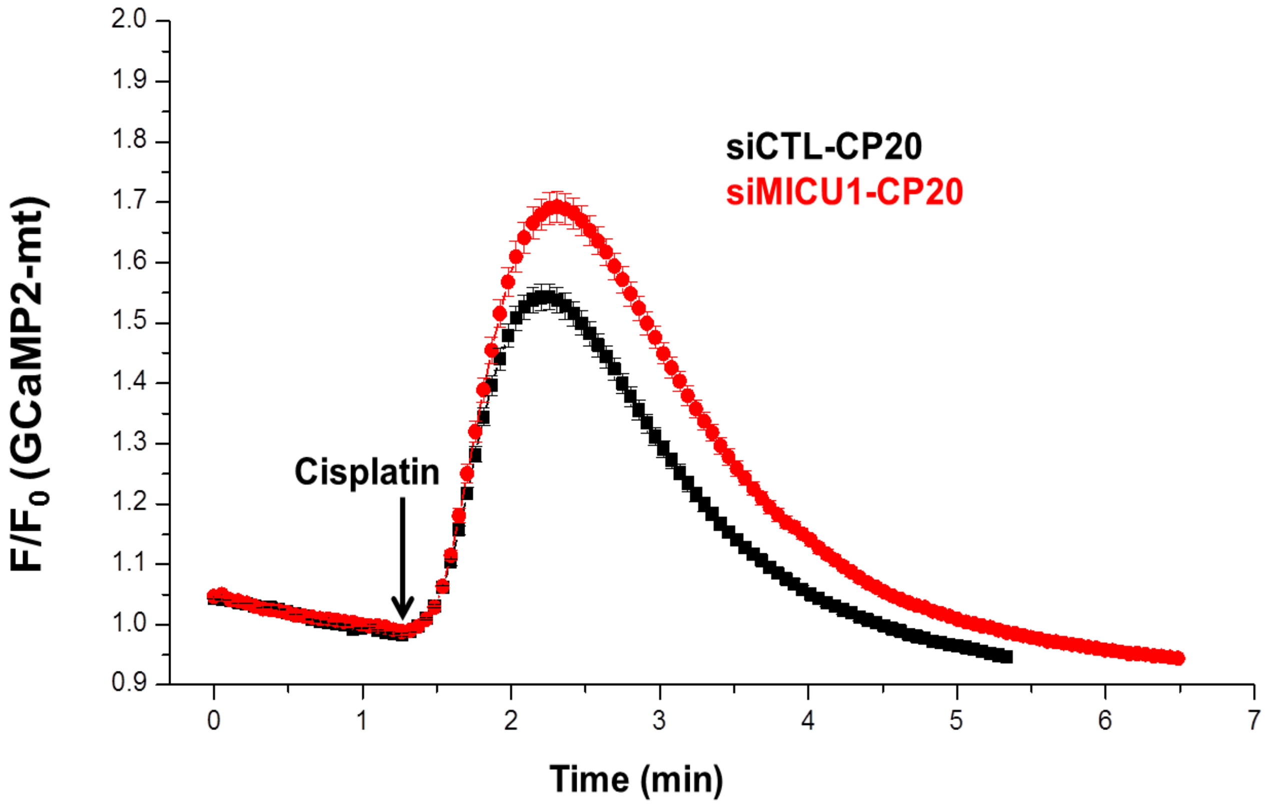

Supplementary Figure 10. MICU1 silencing increases $Ca^{2+}{}_m$. Normalized GCaMP2-mt fluorescence in siRNA-transfected (siCTL-CP20) cells or MICU1 specific siRNA transfected cell (siMICU1-CP20) cells stimulated with cisplatin (10μM) in Ca^{2+} free HBSS buffer. The experiment was repeated three times (total no. of cells quantified: siCTL-CP20 n= 926 and siMICU1-CP20 n= 877).

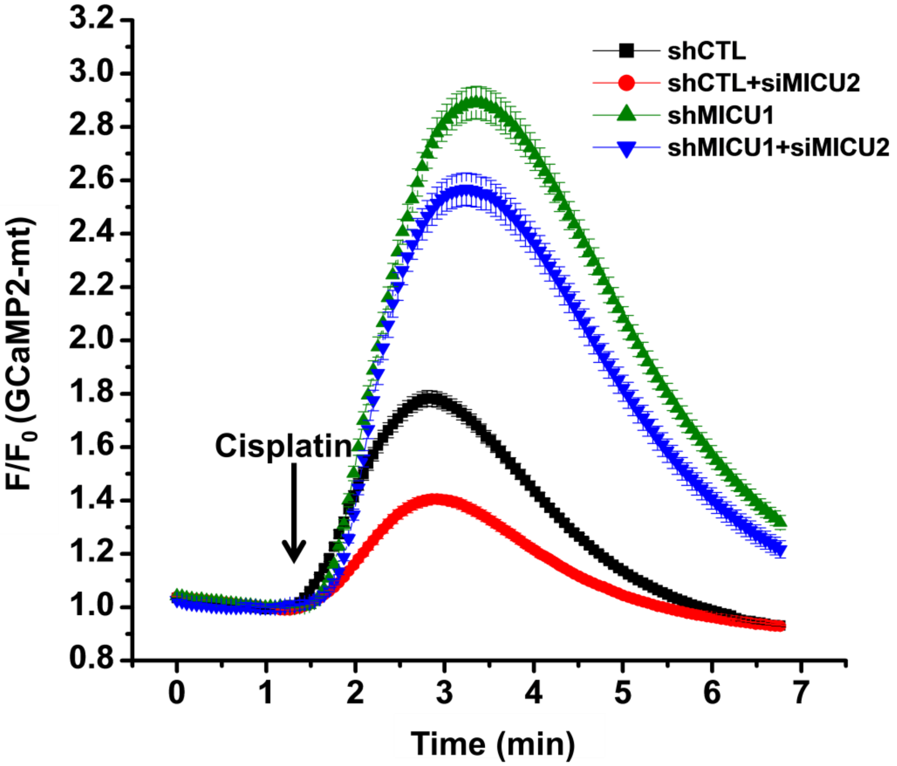

Supplementary Figure 11. MICU2 silencing decreases Ca^{2+}_m. Normalized GCaMP2-mt fluorescence in shRNA-transfected (shCTL-OV90) cells, MICU2 specific siRNA transfected (siMICU2-OV90) cells, shMICU1-OV90 cells or shMICU1-OV90 tranfected with siMICU2 (shMICU1+siMICU2) cells stimulated with cisplatin (10μM) in Ca^{2+} free HBSS buffer. The experiment was repeated twice (total no. of cells quantified: shCTL n=566; shCTL+siMICU2 n=691; shMICU1 n=404 and shMICU1+siMICU2 n=284).

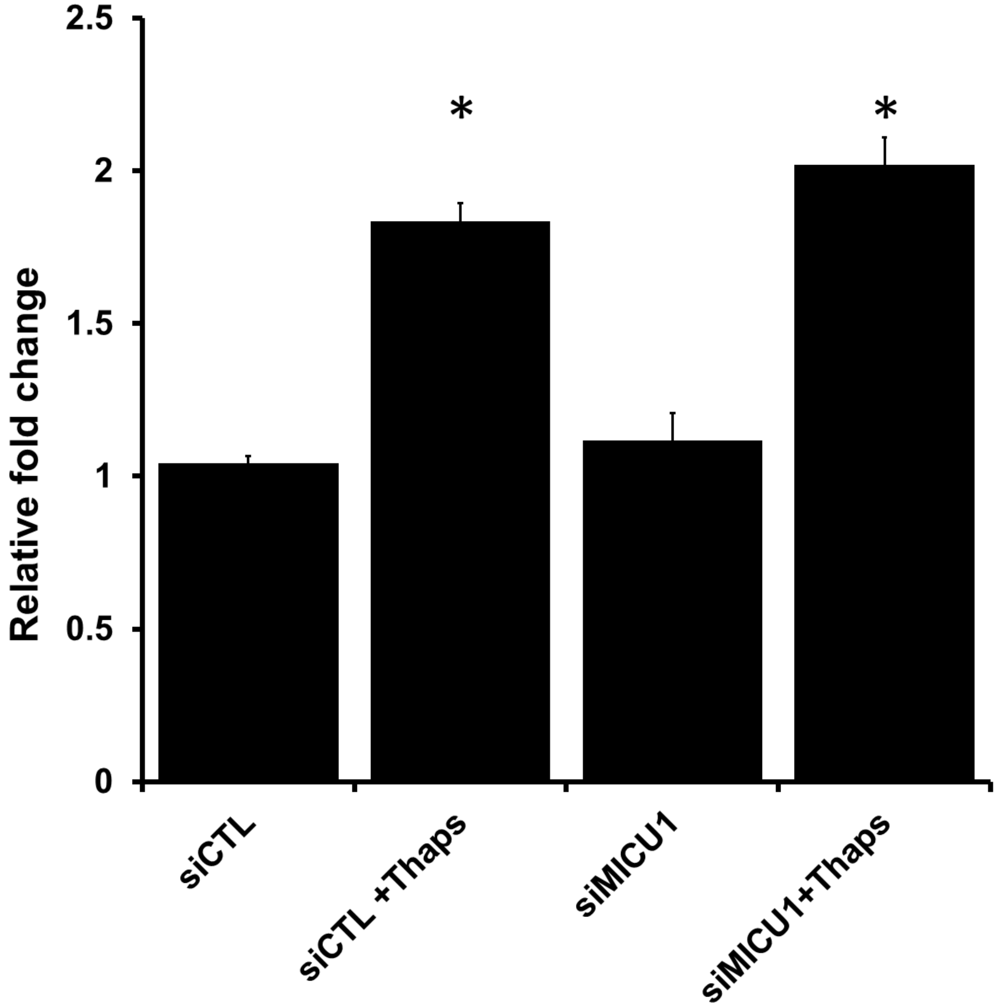

Supplementary Figure 12. Silencing of MICU1 does not affect $[Ca^{2+}]_{ER}$ release. Dose Cisplatin causes similar release of $[Ca^{2+}]_{ER}$ in presence or absence of MICU1 as determined in CP20 cells loaded with calcium indicator dye Fura-2AM (5 µm) and then exposed to Thapsigargin (5 µm) followed by fluorescence measurement Data are expressed as mean ± SD. Comparisons between two groups treated with same dose of Thaps were evaluated. *, $P<0.05$ was statistically significant when compared to respective control. Two-sided Student's t test with unequal variances was used for statistical analysis. All the experiments were repeated independently at least 3 times and in triplicate.

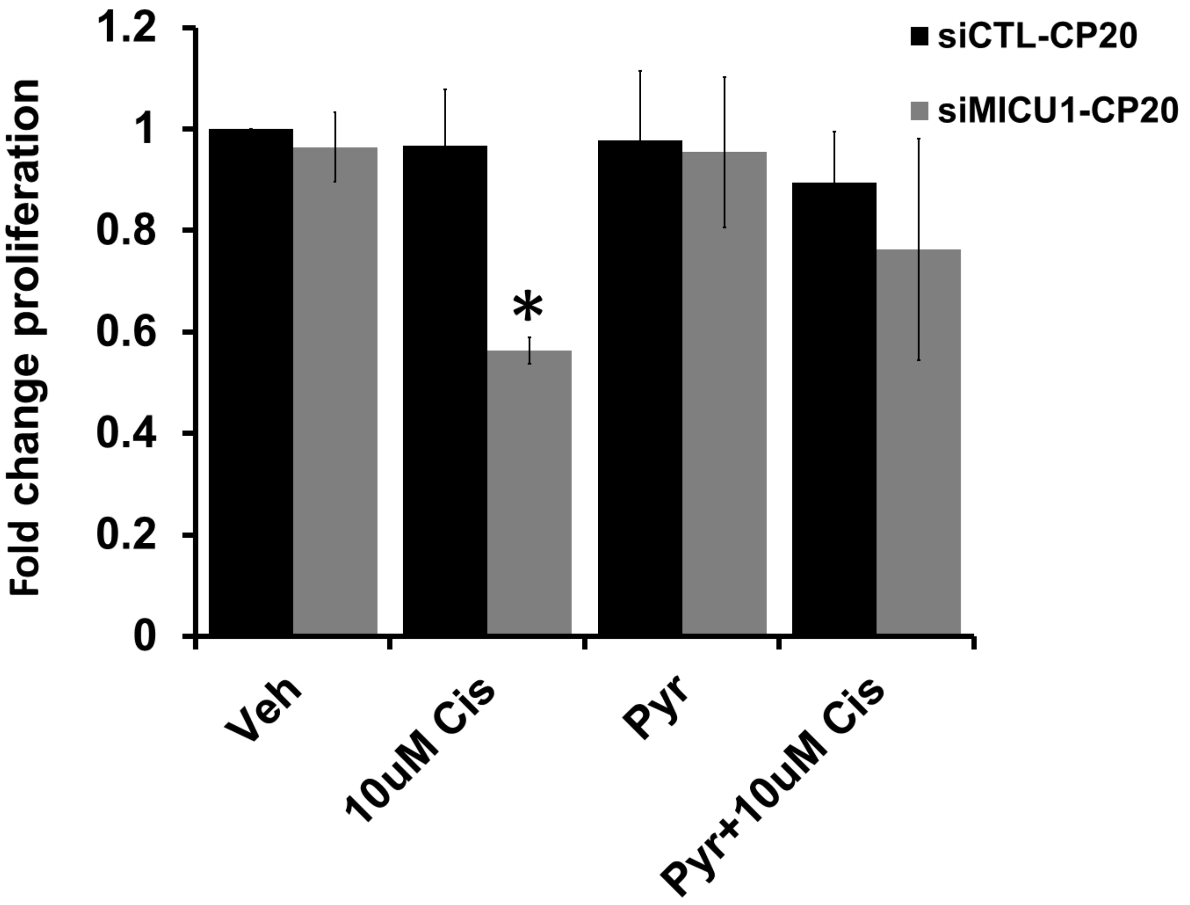

Supplementary Figure 13. ER calcium contributes to cisplatin induced toxicity. CP20 cells were transfected with siCTL or siMICU1 for 24h and $3x10^3$ cells were re-plated in 96-well plates. Post 24h cells were treated with Pyr6 (50nM) for 45 minutes followed by 10 μm cisplatin treatment and after 48 h cell proliferation was determined using CyQUANT® NF Cell Proliferation Assay by measuring the fluorescence (excitation ~485 nm and emission ~530 nm). Fold changes in cell proliferation was evaluated by comparing with vehicle (V) treated siCTL cells. Data are expressed as mean ± SD. Comparisons between two groups treated with same dose of the drug were evaluated. *, P<0.05 was statistically significant when compared to respective control. Two-sided Student's t test with unequal variances was used forstatistical analysis. All the experiments were repeated independently at least 3 times and in triplicate.

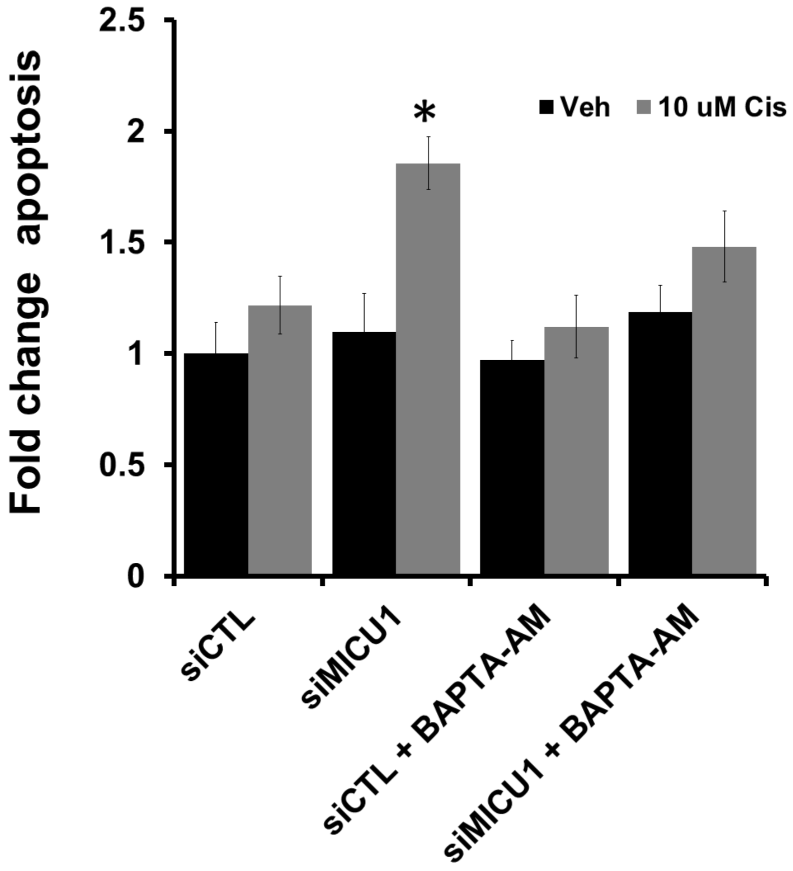

Supplementary Figure 14. ER calcium contributes to cisplatin induced toxicity. CP20 cells ($5X10^5$) were transfected with siCTL or siMICU1 for 24h and cells were re-plated in 96-well plates ($3X10^3$/well). Post 24h, cells were treated with BAPTA-AM (10μM) for 1 hour followed by 10 μm cisplatin treatment. Cell proliferation was determined using CyQUANT® NF Cell Proliferation Assay by measuring the fluorescence (excitation ~485 nm and emission ~530 nm). Fold changes in apoptosis was evaluated by comparing with vehicle (V) treated siCTL cells. Data are expressed as mean ± SD. Comparisons within same groups treated with vehicle or cisplatin were evaluated. *, $P<0.05$ was statistically significant when compared to respective control. Two-sided Student's t test with unequal variances was used for statistical analysis. All the experiments were repeated independently at least 3 times and in triplicate.

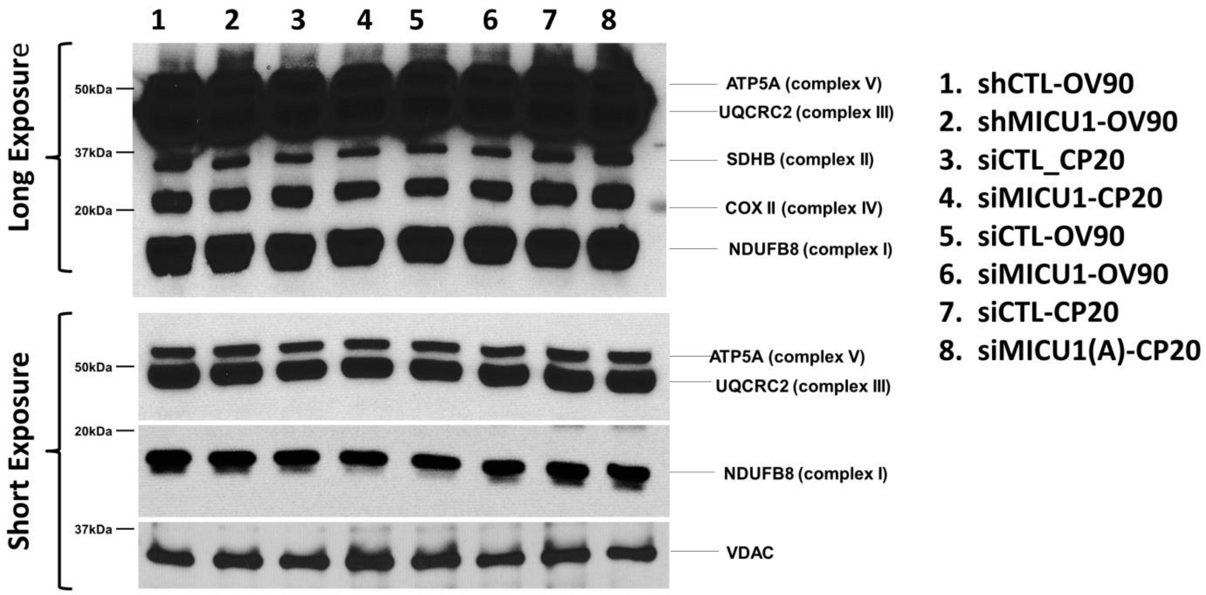

Supplementary Figure 15. Silencing of MICU1 has no effect on the OXPHOS enzyme complexes. The samples are mitochondrial fractions from CP20 and OV90 cells with/without MICU1 silencing. MICU1 gene silencing in OV90 and CP20 cells was achieved by transient siRNA and/or stable shRNA approach. Cells were harvested (48h post transfection for siRNA apporoach) and subjected to differential centrifugation using sucrose gradient to isolate mitochondria enriched fraction. Protein concentration was determined by BCA method and 10µg of protein was subjected to gel electrophoreis using Biorad 4–20% gradient gel and probing with Total OXPHOS Human WB Antibody Cocktail (MitoSciences# MS601) which identifies one representative protein (as indicated) from each electron transport complex I to V . VDAC was used as a loading control. The lower panel represents optimized shorter exposure of the Complex V, III and I immunobands, while a single long exposure for all the immunobands are presented in the top panel.

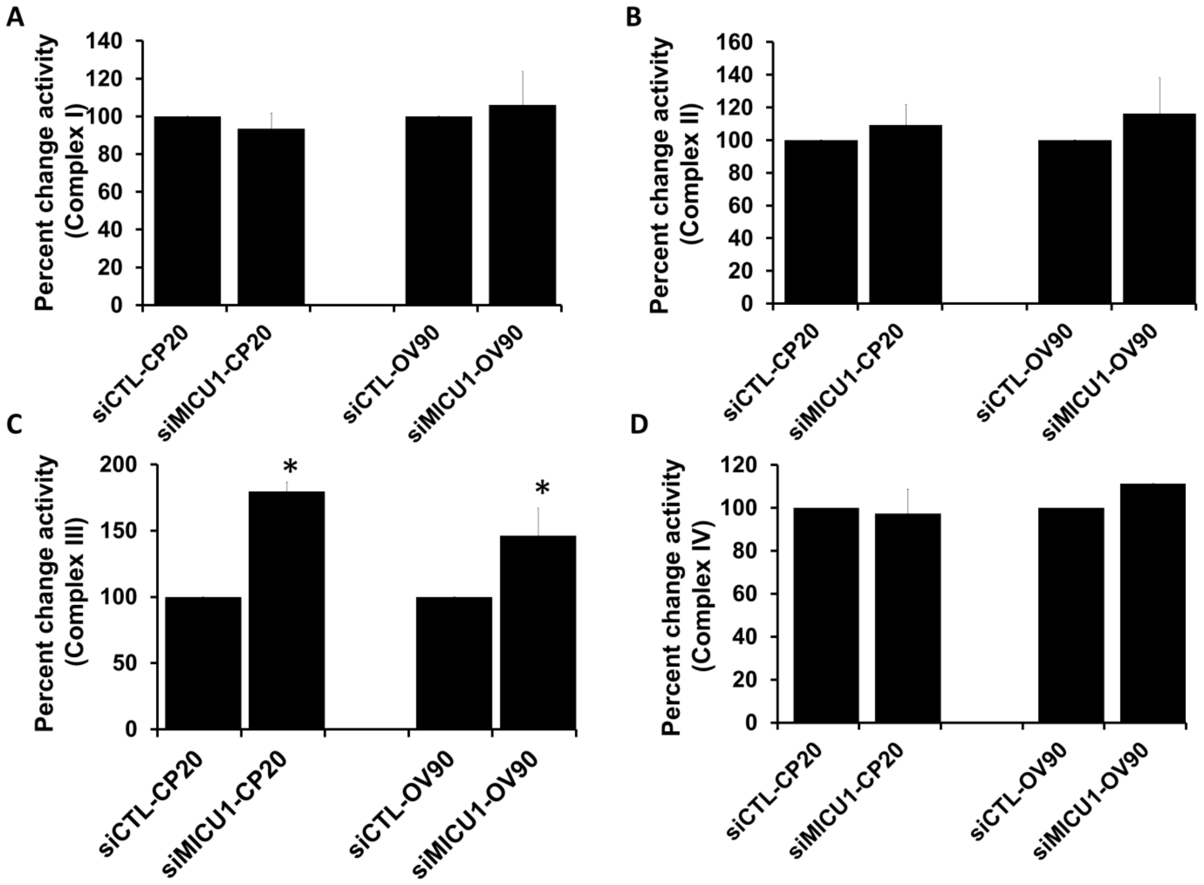

Supplementary Figure 16. Effect of silencing MICU1 on the enzymatic activity of OXPHOS complexes. Mitochondria enriched fraction was obtained, using differential centrifugation with sucrose gradient, from CP20 or OV90 cells transiently transfected with scrambled negative siRNA (siCTL) or MICU1 siRNA (siMICU1). Equal concentration of protein were subjected to the following assays: (A) Complex I Enzyme Activity Assay- Complex I activity was determined by following the oxidation of NADH to NAD^+ and the simultaneous reduction of a dye which leads to increased absorbance at OD=450 nm using a Kit from Abcam (ab109721) . (B) Enzymatic assay for Complex II (succinate dehydrogenase/co-enzyme Q reductase) was performed using MitoCheck® Complex II Activity Assay Kit [Cayman chemical (#700940)] was performed where complex II oxidizes succinate and electrons are passed to an analog of ubiquinone and then on to DCPIP, which, when oxidized, absorbs in the 600 nm range. (C) Enzymatic assay for Complex III (CoQ cytochrome c oxidoreductase) was performed using MitoCheck® Complex II/III Activity Assay Kit [Cayman chemical (#700950)] and measuring\ the reduction of excess cytochrome *c* (550 nm absorbance) as catalyzed by complex III (D) Enzymatic assay for Complex IV (cytochrome c oxidase) was performed using MitoCheck® Complex IV Activity Assay Kit [Cayman chemical (#700990)] and measuring absorbance at 550 nm represnting the oxidation rate of reduced cytochrome c. Data are expressed as mean percent change ± standard deviation (SD). *, $P<0.05$ considered statistically significant. when compared to the respective controls, using two-sided Student’s t-test with unequal variances. All the experiments were repeated independently at least 3 times and in triplicate.

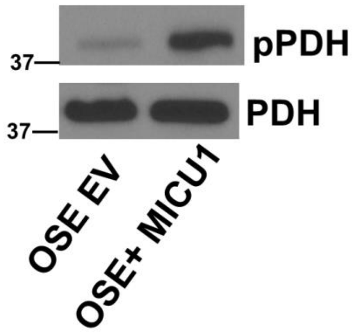

Supplementary Figure 17. Overexpression of MICU1 inactivates PDH. $1X10^6$ Ovarian Surface Epithelial (OSE) cells was transfected with Flag-MICU1 (1 µg) using Lipofectamine 3000 transfection reagent as per the manufacturer's protocol. MICU1-overexprssing OSE cells shows higher expression for phospho PDH (ser293) as determined by immunoblotting. The total PDH level remains unchanged and serves as loading control.

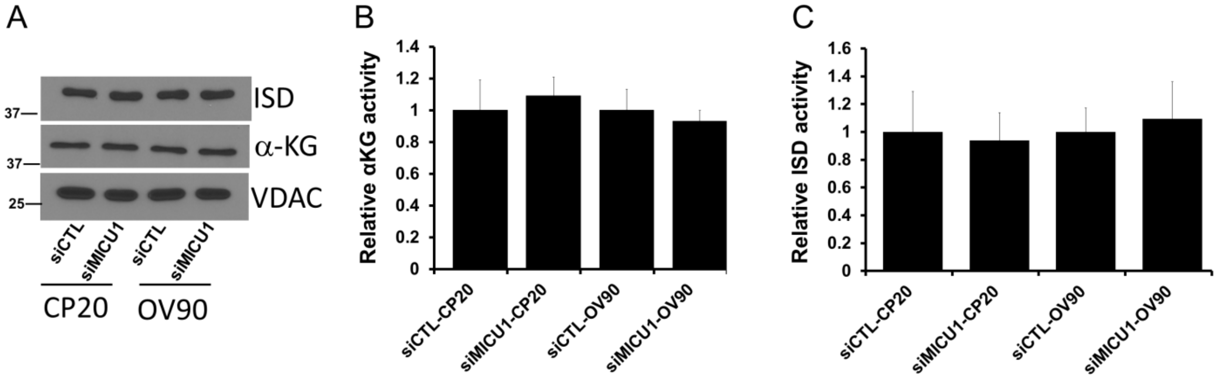

Supplementary Figure 18. Silencing MICU1 doesnot affect alpha-Ketoglutarate and Iso-citrate dehydrogenase. (A) Protein levels of ISD and a-KG were determined by immunoblotting in mitochondria enriched fractions isolated from CP20 or OV90 cells transfected with srambled siRNA (siCTL) or siRNA against MICU1 gene (siMICU1). The total VDAC level remains unchanged and serves as loading control. (B) Alpha-KG activity was measured in CP20 or OV90 cells transfected with siCTL or siMICU1 using Sigma's α-Ketoglutarate Dehydrogenase Activity Colometric Assay kit (MAK189). α-Ketoglutarate Dehydrogenase converts α-Ketoglutarate to an intermediate which interacts with the probe to generate a coloured substance with stong absorbance at 450 nm. (C) ISD activity was measured in CP20 or OV90 cells transfected with siCTL or siMICU1 using Abcam's Isocitrate Dehydrogenase Assay Kit (ab102528, according to the manufacturer's instructions. Isocitrate Dehydrogenase utilizes isocitrate as a specific substrate leading to a proportional color development that was quantified by determining the absorbance of product color at 450 nm in a kinetic mode upto 5 minutes from the stabilization/initiation of the reaction. Data are expressed as mean ± SD. Data is not significant when compared to the respective controls, using two-sided Student's t-test with unequal variance. All the experiments were repeated independently at least 3 times and in triplicate.

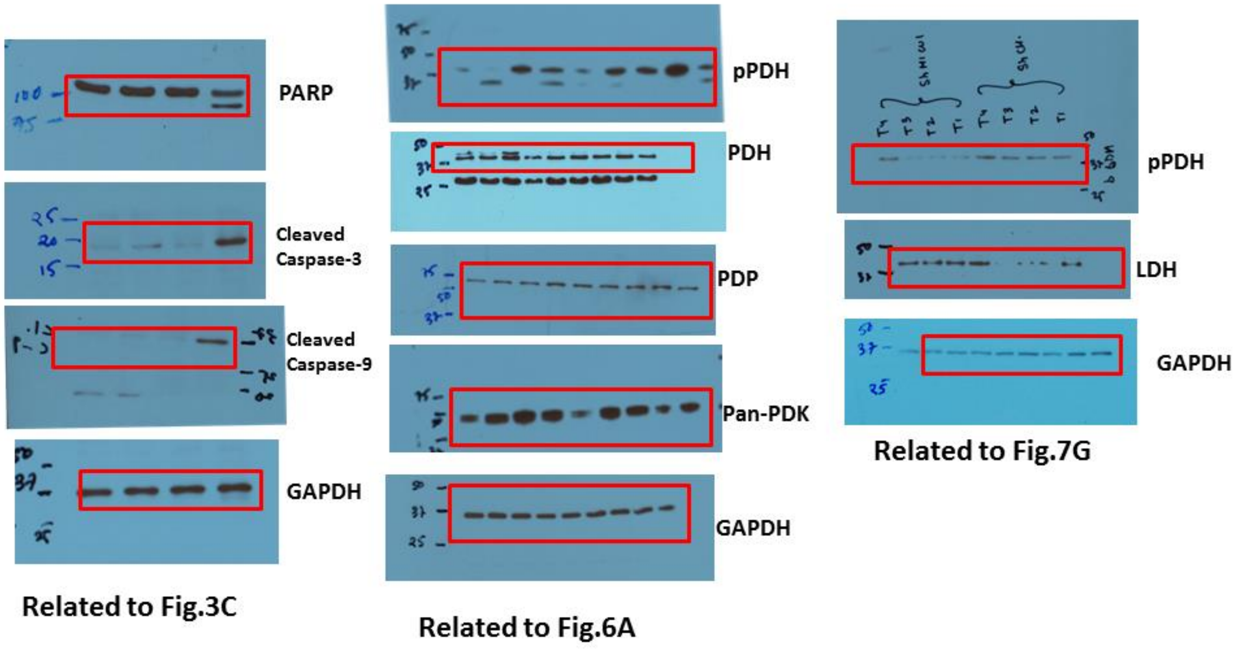

Supplementary Figure 19. Uncropped images of Immunoblots are presented. Reference to the original figures is presented for each blot.

Supplementary Table 1. Patient Cohorts.

	GSE32062	GSE26712
**Number**	260	185
**Age (SD)**	NA	62 (12)
**Stage**		
II	0	0
III	204	144
IV	56	41
**Tumor Grade**		
G2	131	40
G3	129	144
**Surgical Outcome**		
Optimal	103	90
Suboptimal	157	95
**Vital Status**		
Living	132	56
Deceased	110	129
**Follow Up**		
Median (Range)	42 (1-128)	38 (1-164)
**Source**	**GSE32062**	**GSE26712**
**PMID**	**22241791**	**18593951**
**Platform**	**Agilent**	**Affymetrix**

Supplementary Table 2. Correlation between pPDH and MICU1 within each PFS group.

PFS group	Sample size	Spearman correlation coefficient between pPDH and MICU1	p-value*
<6 months	19	0.46	0.048
6-12 months	34	0.46	0.007
>12 months	72	0.53	<0.0001

* The SAS software (version 9.3) was used to compute the p-values, which was based on the treating $(n-2)^{\frac{1}{2}}\left(\frac{r^2}{1-r^2}\right)^{\frac{1}{2}}$ as t-distributed with n-2 degrees of freedom, where r is the sample Spearman correlation.

Supplementary Table 3. Correlation between pPDH and PFS group.

The overall test for group differences was significant (p=0.023, Kruskal-Wallis test). Further pairwise comparisons indicated that the pPDH expression level was significantly higher among '<6mo' compared to '>12mo' group (Bonferroni-adjusted p=0.009).

**Analysis Variable : pPDH**							
**PFS group**	**N Obs**	**N**	**Mean**	**Median**	**Minimum**	**Maximum**	**Std Dev**
<6 mo	19	19	6.66	7.00	3.33	9.00	1.24
6-12 mo	34	34	5.45	6.00	1.33	9.00	2.37
>12 mo	73	72	5.26	5.33	1.00	9.00	2.15

Supplementary Table 4. List of antibodies.

Antibodies	Cat #	Manufacturer	Dilution
MICUI1 (D4P8Q)	2524	CST	1:1000
FLAG	F3165	Sigma	1: 5000
GAPDH	G9545	Sigma	1:5000
OX-PHOS Cocktail	MS601	Mitosciences	1:2500
VDAC	4661S	CST	1:2000
Phospho-PDH (S293)	ABS204	Millipore	1:500
PDH	ab110330	Abcam	1:1000
ISD	ab81653	Abcam	1:1000
α-KG	ab87057	Abcam	1 to 1000
PARP	9532	CST	1 to 2000
Cleaved Caspase 3	9664S	CST	1 to 1000
Cleaved Caspase 9	9501S	CST	1 to 1000
PDP1	ab198261	Abcam	1 to 1000
Pan-PDK	ab115321	Abcam	1 to 1000
LDH	3582	CST	1 to 2000
